# Biofortification of mungbean (*Vigna radiata* L. (Wilczek)) with boron, zinc and iron alters its grain yield and nutrition

**DOI:** 10.1038/s41598-023-30539-6

**Published:** 2023-03-02

**Authors:** Salwinder Singh Dhaliwal, Vivek Sharma, Arvind Kumar Shukla, Manmeet Kaur, Janpriya Kaur, Vibha Verma, Prabhjot Singh, Viliam Barek, Ahmed Gaber, Akbar Hossain

**Affiliations:** 1 Department of Soil Science, Punjab Agricultural University, Ferozepur Rd, Ludhiana, 141027 Punjab India; 2 Indian Institute of Soil Science, Berasia Rd, Navi Bagh, Bhopal, 462038 Madhya Pradesh India; 3 Department of Water Resources and Environmental Engineering, Faculty of Horticulture and Landscape Engineering, Slovak University of Agriculture, Nitra, Tr. A. Hlinku 2, 949 01 Nitra, Slovakia; 4 Department of Biology, College of Science, Taif University, P.O. Box 11099, Taif, 21944 Saudi Arabia; 5 Bangladesh Wheat and Maize Research Institute, Dinajpur, 5200 Bangladesh

**Keywords:** Biochemistry, Plant sciences

## Abstract

Mungbean [*Vigna radiata* L. (Wilczek)] is considered as an extremely nutritious crop possessing a high level of micronutrients, but their low bioavailability in the crop leads to micronutrient malnutrition in humans. Therefore, the present study was conducted to investigate the potential of nutrients viz. boron (B), zinc (Zn) and iron (Fe) biofortification on productivity, nutrient concentration and uptake as well as the economics of mungbean cultivation. In the experiment, the various combinations of RDF with ZnSO_4_.7H_2_O (0.5%), FeSO_4_.7H_2_O (0.5%) and borax (0.1%) were applied to mungbean variety ML 2056. The combined foliar application of Zn, Fe and B was highly efficient in increasing the yield of grain as well as straw in mungbean exhibiting maximum values i.e. 944 kg ha^−1^ and 6133 kg ha^−1^, respectively. Similar results for B, Zn and Fe concentration in grain (27.3 mg kg^−1^, 35.7 mg kg^−1^ and 187.1 mg kg^−1^, respectively) and straw (21.1 mg kg^−1^, 18.6 mg kg^−1^ and 376.1 mg kg^−1^, respectively) of mungbean were observed. Also, uptake of Zn and Fe by grain (31.3 g ha^−1^ and 164.4 g ha^−1^, respectively), as well as straw (113.7 g ha^−1^ and 2295.0 g ha^−1^, respectively), was maximum for the above treatment. Whereas, the B uptake was found to enhance significantly through the combined application of B, Zn and Fe, where the values 24.0 g ha^−1^ and 128.7 g ha^−1^ corresponded to grain and straw, respectively. Thus, combined use of ZnSO_4_.7H_2_O (0.5%) + FeSO_4_.7H_2_O (0.5%) and borax (0.1%) significantly improved the yield outcomes, the concentration of B, Zn and Fe, uptake and economic returns of mungbean cultivation to alleviate the B, Zn and Fe deficiency.

## Introduction

Legumes are considered an essential source of protein which is consumed globally. Among different legumes, mungbean [*Vigna radiata* L. (Wilczek)] is a highly nutritious as well as inexpensive protein and vitamin source^1,2^, which can be grown two times in one year, firstly in the Kharif season during July to October and secondly in summer during March to June, as it involves shorter time span^3^. It grows best in a moist climate, although its sowing can be done in an area where the availability of water is limited^4,5^. Besides this, mungbean has a vital biological nitrogen fixation function that improves soil health by modifying chemical, physical, and biological qualities, as well as increasing soil nitrogen content.

Micronutrient deficiency in soils and crops, such as B, Zn, and Fe insufficiency has resulted in severe effects such as decreased yield and low micronutrient concentration in crops, leading to micronutrient malnutrition in humans and animals^6,7^. Boron is an essential element for basic plant processes like photosynthesis, protein and chlorophyll synthesis^8^. It is important for root growth and carbohydrate synthesis, and its absence causes stunting and deformation of the growing tips, which can lead to tip mortality, brittle foliage, and yellowing of lower leaf tips. In humans and animals, B deficiency causes an immunological function to deteriorate, as well as increased mortality risk, cell damage, and toxicity^9^. Additionally, Zn is a trace element which is considered crucial as it possesses antioxidant properties and is required for proper growth, immune system development, enzyme activation, and neurobehavioral development^10^. Its lack in the diet may result in serious health-related issues, such as stunted growth in children, increased illness susceptibility, poor birth outcomes, and harm to the brain and immune system^11^.

In addition to this, Fe plays an outstanding role in plant respiration, photosynthesis, sulphur absorption, and nitrogen-fixing. It acts as an important protein constituent which helps in transporting oxygen and regulating cell growth along with differentiation. Fe deficit diet leads to limited oxygen delivery to cells resulting in fatigue, lowered immunity, and an increased risk of blood anaemia^12–14^. Further, deficiency of Fe may lead to chlorosis in crops along with the deterioration of produce^15–17^.

To combat micronutrient deficiency, a variety of traditional interventions have been applied, including dietary supplementation, food fortification, and dietary diversification^18^. Due to a lack of infrastructure, these strategies were found to be unsuccessful. In this view, an alternate key to malnutrition is biofortification suggested by various earlier research findings. Biofortification helps in enhancing the concentration of micronutrients in a crop using specialized techniques such as plant breeding and agronomic procedures^19^. Further, agronomic biofortification through foliar sprays, seed priming and soil treatments are considered convenient ways to improve the nutritional content in the crop.

Agronomic biofortification is a short-term strategy for increasing micronutrient concentrations, but it is easier and more feasible to achieve as compared to breeding^20^. It could provide a quick fix for mineral deficits while also serving as a supplement to ongoing breeding programs^21^. Among different types of biofortification techniques, foliar application is considered as the best way of increasing the micronutrient level in crops, as nutrients are directed towards the leaves at suitable growth stages^22^. The quick absorption encourages nutrient translocations in edible grain sections and prevents nutrient loss in the environment. Furthermore, it encourages plant development even under less favourable weather circumstances.

Several researchers have been probing the effect of enhancing bioavailable B, Zn and Fe in various legume crops through foliar application. Biofortification of Zn has been found to escalate the grain yield along with the concentration of Zn in mungbean^23^. Another study demonstrated the priming of Zn seed to enhance the growth and yield in mungbean through biofortification^24^. Additionally, biofortification of mungbean by using Fe-enriched biochar, compost and poultry manure resulted in increased growth as well as yield significantly^25^. Keeping this in view, the present work aimed to assess the influence of the foliar application of B, Zn and Fe on yield, concentration and absorption, to demonstrate its potential for biofortification as a food source that provides nutritional security to mungbean consumers.

## Materials and methods

### Site specification and characteristics

The location of the two-year study included the farm where the experiment was conducted, Department of Soil Science, Punjab Agricultural University (PAU), Ludhiana, Punjab (30° 56′ N, 75° 52′ E, and 247 m above mean sea level) in the Indo-Gangetic plains of north-western India during the Kharif season (June-October). The experimental soil possessed a pH of 7.21, an EC of 0.34dS m^−1^, and an OC of 0.31%. Micronutrient levels in soil were initially 1.16, 0.65, 4.86, and 3.91 mg kg^−1^ in the case of Zn, Cu, Fe, and Mn, respectively. The region exhibited a subtropical climate along with hot, rainy summers as well as dry winters. The annual rainfall ranges between 400 and 600 mm and the months of July to September receive the majority of the rainfall, which is around 70%.

### Treatment details

The study was performed following a randomized block design along with three replications of eight treatments over two years (Table 1). Different combinations of ZnSO_4_.7H_2_O (0.5%), FeSO_4_.7H_2_O (0.5%) and borax (0.1%) were applied to mungbean through foliar application. The high-yielding and disease-resistant variety of mungbean ML 2056 was used in the present study which is developed by PAU, Ludhiana in the year 2016. The average yield potential of the variety is 11.5 q/ha with green and medium bold shining grains having good cooking quality. The seed was purchased from the Department of Plant Breeding and Genetics, PAU, Ludhiana. The experimental field was subjected to two ploughings followed by planking. During planting, the recommended dose of N: 11 kg ha^−1^, P_2_O_5_: 100 kg ha^−1^ was applied as a basal through urea and diammonium phosphate, respectively. The sowing of mungbean was done during the first week of June using the drill method with row to row distance of 22.5 cm and plot size of 5.0 m $\times$ 4.0 m, whereas the harvesting was performed in the first week of October.

**Table 1:** Treatment details of the experimental field at the time of sowing of lentil.

S. no	Treatments
T_1_	RDF control
T_2_	RDF + FeSO_4_. 7H_2_O (0.5%) foliar spray 40 DAS
T_3_	RDF + ZnSO_4_. 7H_2_O (0.5%) foliar spray 40 DAS
T_4_	RDF + Borax (0.1%) foliar spray 40 DAS
T_5_	RDF + FeSO_4_. 7H_2_O (0.5%) + ZnSO_4_. 7H_2_O (0.5%) 40 DAS
T_6_	RDF + FeSO_4_. 7H_2_O (0.5%) + Borax (0.1%) 40 DAS
T_7_	RDF + ZnSO_4_. 7H_2_O (0.5%) + Borax (0.1%) 40 DAS
T8	RDF + ZnSO_4_. 7H_2_O (0.5%) + FeSO_4_. 7H_2_O (0.5%) + Borax (0.1%) 40 DAS

*RDF* recommended dose of fertilizers (N: 11 kg ha^−1^, P_2_O_5_: 100 kg ha^−1^).

### Harvesting and analysis

When the plants reached physiological maturity, they were manually harvested, and grain, as well as straw samples, were collected for examination. To measure the dry weight of different components of plant, the samples were air-dried before drying in an oven at 65 °C for 48 h. A mechanical grinder was used to grind oven-dried plant samples to a fine powder. On an electric hot plate, the grounded samples of straw and grain weighing 1.0 g and 0.5 g, respectively, were subjected to the digestion using a mixture of di-acid i.e. HNO_3_ and HClO_4_ acid in a 3:1 ratio^26^. The micronutrients such as Fe, Mn, Zn, and Cu in digested extracts of the plant were measured using an atomic absorption spectrophotometer (Model AAS 240 FS, Company Varian, Germany). It is certified that all methods were performed according to the relevant guidelines and regulations.

### Boron, zinc and iron use efficiency indices

The calculation for the mobilization efficiency index (MEI) involved the following equation:

$$ {\text{MEI}} = \frac{{{\text{Nutrient}}\,\,{\text{concentration}}\,\,{\text{in}}\,\,{\text{grain}}\,{\text{(mg}}\,\,{\text{kg}}^{ - 1} )}}{{{\text{Nutrient}}\,\,{\text{concentration}}\,\,{\text{in}}\,\,{\text{straw}}\,{\text{(mg}}\,\,{\text{kg}}^{ - 1} )}} $$

The determination of physiological efficiency of B, Zn and Fe viz. (PE_B_), (PE_Zn_), (PE_Fe_), apparent recovery efficiency (ARE-B), (ARE-Zn), (ARE-Fe) and mobilization efficiency index (MEI-B), (MEI-Zn), (MEI-Fe) of B, Zn and Fe was done through the equations given below^27^.

$$ \begin{gathered} {\text{PE }} = {\text{ Y}}_{{\text{t}}} {-}{\text{ Y}}_{{\text{c}}} /{\text{NU}}_{{\text{t}}} - {\text{ NU}}_{{\text{c}}} \left( {{\text{kg ha}}^{{ - {1}}} } \right) \hfill \\ {\text{ARE}} = {\text{ NU}}_{{\text{t}}} - {\text{ NU}}_{{\text{c}}} /{\text{Nutrient applied }}\left( {{\text{kg ha}}^{{ - {1}}} } \right) \times 100 \hfill \\ \end{gathered} $$

where, Y_t_ and Y_c_ denote grain yield (kg ha^−1^) of mungbean in B, Zn and Fe fertilized plots as well as in control, respectively; NU_t_ and NU_c_ denote the total nutrient (B, Zn, Fe) uptake (kg ha^−1^) of mungbean in B, Zn and Fe fertilized plots as well as in control, respectively.

### Economic analysis

The cost of fertilizer in the United State Dollar (USD) ha^−1^ for various treatments in the experiment was worked out separately, considering the prevailing prices of fertilizers in USD at the time of their use. Gross return (value of additional yield) was calculated based on the MSP (price for minimum support) of the mungbean by the Indian government during the years of study. Net return (USD ha^−1^) was calculated by subtracting the cost of fertilizer from the gross return as given below.

$$ {\text{Net}}\,{\text{Return}}\,\,{\text{(USD}}\,\,{\text{ha}}^{ - 1} ) = ({\text{Gross}}\,{\text{return}} - {\text{Cultivation}}\,\,{\text{cost}})({\text{USD}}\,\,{\text{ha}}^{ - 1} ) $$

B:C ratio was calculated from the equation:

$$ {\text{B:C}}\,\,{\text{ratio}} = \frac{{{\text{Gross}}\,\,{\text{return}}}}{{{\text{Cultivation}}\,\,{\text{cost}}}} $$

### Statistical analysis

Two-year data were analyzed statistically using SPSS version 16.0 (SPSS Inc., Chicago, USA) packages. All parameters were studied using a one-way analysis of variance (ANOVA) for comparing means and differences among the treatments by using the Duncan Multiple Range Test (DMRT) at a 0.05 probability level.

### Ethical approval

It is certified that all methods were performed according to the relevant guidelines and regulations.

## Results

### Impact of foliar application of B, Zn and Fe on grain and straw yield of mungbean

The two-year mean data demonstrated that the application of B, Zn and Fe posed a significant impact on the yield of grain as well as straw in mungbean (Table 2). The minimum value of grain and straw yield was observed in treatment T1 with mean values of 726 kg ha^−1^ and 4685 kg ha^−1^, respectively. Additionally, combined treatment T5 (FeSO_4_. 7H_2_O (0.5%) + ZnSO_4_. 7H_2_O (0.5%) at 40 DAS) was found to be less effective in enhancing the grain (833 kg ha^−1^) and straw (5747 kg ha^−1^) yield in comparison to the treatments T6 (FeSO_4_. 7H_2_O (0.5%) + borax (0.1%) at 40 DAS) and T7 (ZnSO_4_. 7H_2_O (0.5%) + borax (0.1%) at 40 DAS) exhibiting the grain yields of 873 kg ha^−1^ and 899 kg ha^−1^ and straw yields of 5790 kg ha^−1^ and 5929 kg ha^−1^, respectively (Table 2). But, treatment T5 was not statistically different from treatment T6 (873 kg ha^−1^) in grain yield, and with T6 (5790 kg ha^−1^) and T7 (5929 kg ha^−1^) in straw yield. Thus, the presence of B caused extreme enhancement in the yield of grain as well as straw in mungbean. This result was further confirmed by the foliar application of ZnSO_4_. 7H_2_O (0.5%) + FeSO_4_. 7H_2_O (0.5%) + borax (0.1%) at 40 DAS (treatment T8) which possessed the highest yields of grain and straw with values of 944 kg ha^−1^ and 6133 kg ha^−1^, respectively. Treatment T8 was statistically at par with treatment T7 (899 kg ha^−1^) during the first-year study in the case of grain yield. Whereas in the case of straw yield, treatment T8 was not statistically different from treatment T6 (5790 kg ha^−1^) during the second year.

**Table 2:** Impact of B, Zn and Fe biofortification on grain and straw yield of Mungbean.

Treatments	Grain yield (kg ha^−1^)			Straw yield (kg ha^−1^)		
	Year1	Year II	Mean	Year1	Year II	Mean
T1	810^c^	642^f^	726^g^	4170^e^	5200^e^	4685^e^
T2	890^b^	678^e^	784^f^	4380^e^	5580^d^	4980^d^
T3	920^b^	727^d^	824^ef^	4680^d^	5748^cd^	5214^c^
T4	880^b^	718^d^	799^ef^	4590^d^	5732^d^	5161^c^
T5	890^b^	775^c^	833^de^	5510^c^	5983^ab^	5747^b^
T6	910^b^	835^b^	873^cd^	5580^c^	6000^a^	5790^b^
T7	990^a^	807^bc^	899^bc^	5940^b^	5918^bc^	5929^b^
T8	1010^a^	878^a^	944^a^	6160^a^	6105^a^	6133^a^
CV (%)	0.163	0.176	0.168	0.021	0.026	0.024
LSD (P ≤ 0.05)	47	33	41	214	170	192

Treatment details are referred to in Table 1. By Duncan’s multiple range test, the values with similar letter(s) in superscript do not differ significantly at the 5% level.

### Impact of foliar application of B, Zn and Fe on concentration in grain and straw of mungbean

The mean of two-year data for grain, as well as straw B, Zn and Fe concentration in mungbean is due to the foliar use of B, Zn and Fe, which is presented in Table 3. All B, Zn and Fe combinations resulted in increased concentration of micronutrients in grain as well as straw yield in comparison to control. However, the foliar application of Zn and Fe presented a significant impact on their concentration in grain as well as straw of mungbean, whereas, no significant impact of B was observed on its concentration in grain and straw of mungbean, possibly resulting from a relatively large field variation. The results of grain B concentration in mungbean suggested that treatment T8 (27.3 mg kg^−1^) showed more enhancements in concentration as compared to treatment T1 in which the minimum value of B concentration was observed (25.4 mg kg^−1^). The results of grain Zn concentration in mungbean demonstrated that the maximum Zn concentration was observed in treatment T8 (35.7 mg kg^−1^), which was statistically at par with treatments T2 (33.3 mg kg^−1^), T5 (34.5 mg kg^−1^) and T6 (33.4 mg kg^−1^) (Table 3). However, the concentration of Zn in grain was recorded to be minimum in treatment T1 (30.5 mg kg^−1^) which was not statistically different from treatments T3 (34.9 mg kg^−1^) and T4 (33.9 mg kg^−1^). Thus, the foliar application of ZnSO_4_. 7H_2_O (0.5%) + FeSO_4_. 7H_2_O (0.5%) + borax (0.1%) was most efficient in enhancing the Zn concentration in the grain of mungbean. Similarly, the mean concentration of Fe in grain was found to be maximum for treatment T8 (187.1 mg kg^−1^) which was statistically at par with treatments T2 (177.5 mg kg^−1^), T5 (188.4 mg kg^−1^) and T6 (184.3 mg kg^−1^) and the minimum Fe concentration in grain was observed in T1 (103.1 mg kg^−1^).

**Table 3:** Impact of foliar application of B, Zn and Fe on concentration in grain and straw.

Treatments	Grain concentration (mg kg^−1^)			Straw concentration (mg kg^−1^)		
	Boron	Zinc	Iron	Boron	Zinc	Iron
T1	25.4^a^	103.1^d^	30.5^c^	20.0^a^	12.2^d^	186.9^d^
T2	28.4^a^	177.5^ab^	33.3^b^	20.6^a^	14.8^c^	297.4^bc^
T3	27.6^a^	127.1^cd^	34.9^ab^	21.3^a^	18.4^a^	252.8^cd^
T4	29.4^a^	114.8^cd^	33.9^ab^	22.2^a^	16.2^bc^	242.6^d^
T5	26.6^a^	188.4^a^	34.5^ab^	21.6^a^	17.3^ab^	347.5^ab^
T6	29.7^a^	184.3^a^	33.4^b^	21.1^a^	15.1^c^	317.5^b^
T7	30.4^a^	143.2^bc^	35.1^ab^	23.2^a^	18.4^a^	265.7^c^
T8	27.3^a^	187.1^a^	35.7^a^	21.1^a^	18.6^a^	376.1^a^
CV	0.212	0.077	0.217	0.204	0.095	0.062
LSD (P = 0.05)	NS	37.6	1.9	NS	2.0	50.2

Treatment details are referred to in Table 1. By Duncan’s multiple range test, the values with similar letter(s) in superscript do not differ significantly at the 5% level.

The average results of the two-year study concerning B, Zn and Fe concentration in the straw of mungbean after the foliar use of B, Zn and Fe are presented in Table 3. Zn and Fe application showed significant enhancement in grain and straw yield of mungbean, whereas, no significant impact of B was observed. Treatment T8 (21.1 mg kg^−1^) showed a remarkable increase in B concentration in the straw of mungbean, whereas, the lowest concentration of B in straw was found in treatment T1 (20.0 mg kg^−1^). However, treatment T8 exhibited a maximum Zn concentration of 18.6 mg kg^−1^ which was not statistically different from treatments T3 (18.4 mg kg^−1^), T5 (17.3 mg kg^−1^) and T7 (18.4 mg kg^−1^), whereas, the minimum straw Zn concentration was found in T1 (12.2 mg kg^−1^). Thus, the foliar application of ZnSO_4_. 7H_2_O (0.5%) + FeSO_4_. 7H_2_O (0.5%) + borax (0.1%) was highly efficient in increasing Zn concentration in the straw of mungbean. Similarly, the mean concentration of Fe in straw was found to be maximum for treatment T8 (376.1 mg kg^−1^) which was not statistically different from treatment T5 (347.5 mg kg^−1^). The lowest concentration of Fe in straw was observed in treatment T1 (186.9 mg kg^−1^) which was not statistically different from treatments T3 (252.8 mg kg^−1^) and T4 (242.6 mg kg^−1^).

### Impact of foliar application of B, Zn and Fe on their uptake in grain and straw of mungbean

Boron, Zn and Fe uptake by grain, as well as straw in mungbean, significantly enhanced with singular and joint use of B, Zn and Fe as shown in Table 4. Treatment T8 exhibited enhanced B uptake in grain with the value of 24.0 g ha^−1^, which was not statistically different from treatments T6 (24.8 g ha^−1^) and T7 (24.6 g ha^−1^). The lowest B uptake by mungbean was found in treatment T1 (16.3 g ha^−1^), which was statistically at par with treatment T2 (19.2 g ha^−1^). On the other hand, the mean of the two-year data for grain Zn uptake in mungbean demonstrated that the maximum Zn uptake was observed in treatment T8 (31.3 g ha^−1^) and the minimum was found in T1 (19.6 g ha^−1^). Additionally, the highest uptake of Fe in grain was found in treatment T8 (164.4 g ha^−1^) which was not statistically different from treatments T5 (146.0 g ha^−1^) and T6 (153.9 g ha^−1^); whereas, treatment T1 possessed minimum Fe uptake (66.2 g ha^−1^) in the grain of mungbean, which was statistically at par with treatments T3 (92.0 g ha^−1^) and T4 (82.3 g ha^−1^). Overall, the foliar application of treatment T8 possessed the maximum potential for increasing grain Zn and Fe uptake in mungbean.

**Table 4:** Impact of foliar application of B, Zn and Fe on the uptake in grain and straw.

Treatments	Uptake in grain (g ha^−1^)			Uptake in straw (g ha^−1^)		
	Boron	Zinc	Iron	Boron	Zinc	Iron
T1	16.3^e^	19.6f.	66.2^d^	101.2^d^	61.7^e^	946^d^
T2	19.2^de^	22.6^e^	120.4^bc^	113.7^c^	82.1^d^	1647^c^
T3	20.0^d^	25.3^ cd^	92.0^ cd^	122.5^b^	105.5^ab^	1453^c^
T4	21.1^bcd^	24.3^de^	82.3^d^	127.3^b^	93.0^bcd^	1389^c^
T5	20.6^c^	26.8^bc^	146.0^ab^	129.2^ab^	103.6^abc^	2081^b^
T6	24.8^a^	27.9^b^	153.9^a^	127.8^b^	91.7^ cd^	1917^b^
T7	24.6^ab^	28.3^b^	115.2^c^	136.5^a^	108.2^a^	1559^c^
T8	24.0^abc^	31.3^a^	164.4^a^	128.7^ab^	113.7^a^	2295^a^
CV	0.165	0.206	0.225	0.094	0.071	0.072
LSD (P = 0.05)	3.5	2.0	28.8	8.5	13.3	275

Treatment details are referred to in Table 1. By Duncan’s multiple range test, the values with similar letter(s) in superscript do not differ significantly at the 5% level.

The average uptake of B, Zn and Fe by straw in mungbean in both years as affected by sole and joint application of B, Zn and Fe has been presented in Table 4. Mean data suggested that treatment T8 was found to be effective in enhancing B uptake in straw with the value of 128.7 g ha^−1^, which was not statistically different from treatments T5 (129.2 g ha^−1^) and T7 (136.5 g ha^−1^). Whereas, both singular and joint application of Zn and Fe enhanced their uptake in straw as compared to the control. However, the maximum value for Zn and Fe uptake by straw was observed in T8 (113.7 g ha^−1^ and 2295.9 g ha^−1^, respectively) and minimum uptake was observed in control i.e. treatment T1 (61.7 and 946.0 g ha^−1^, respectively). Thus, the foliar application of T8 was most impactful in augmenting the uptake of Zn and Fe in the straw of mungbean.

### Impact of foliar application of B, Zn and Fe on efficiency indices of mungbean

The results of Table 5 demonstrated that the MEI-B was highest in treatment T6 (1.41) and lowest in treatment T1 (1.27). Whereas, the maximum values for MEI-Zn and MEI-Fe were observed in treatments T1 (2.50) and T2 (0.60), respectively and the minimum values were found in treatments T3 (1.90) and T4 (0.47), respectively. The results of PE-B were highest in treatment T8 (45.2), whereas PE-Zn and PE-Fe were the maximum in treatment T4 with values of 33.3 and 2.70, respectively. Minimum values of PE-B, PE-Zn and PE-Fe were found in treatments T5 (19.6), T5 (12.9) and T2 (0.44), respectively. Additionally, the ARE-B, ARE-Zn and ARE-Fe were highest in treatment T4 with values of 24.8, 29.6 and 269.6, respectively. Whereas, the minimum values for ARE-B, ARE-Zn and ARE-Fe were observed in treatments T8 (1.09), T5 (3.64) and T3 (71.9), respectively.

**Table 5:** Impact of foliar application of B, Zn and Fe on micronutrient use efficiencies by mungbean.

Treatments	Mobilization efficiency			Physiologicalefficiency			Apparent Recovery efficiency		
	Boron	Zinc	Iron	Boron	Zinc	Iron	Boron	Zinc	Iron
T1	1.27	2.50	0.55	–	–	–	–	–	–
T2	1.38	2.25	0.60	20.1	13.2	0.44	2.88	4.32	147.9
T3	1.30	1.90	0.50	31.2	15.8	1.54	2.80	8.48	71.9
T4	1.32	2.09	0.47	38.7	33.3	2.70	24.8	29.6	269.6
T5	1.23	1.99	0.54	19.6	12.9	0.56	1.12	3.64	98.4
T6	1.41	2.21	0.58	36.2	33.1	1.31	3.60	3.87	141.2
T7	1.31	1.91	0.54	35.5	28.0	2.52	5.46	7.20	79.3
T8	1.29	1.92	0.50	45.2	25.0	1.18	1.09	4.21	99.3

Treatment details are referred to in Table 1.

### Economic analysis

**Figure 1:**
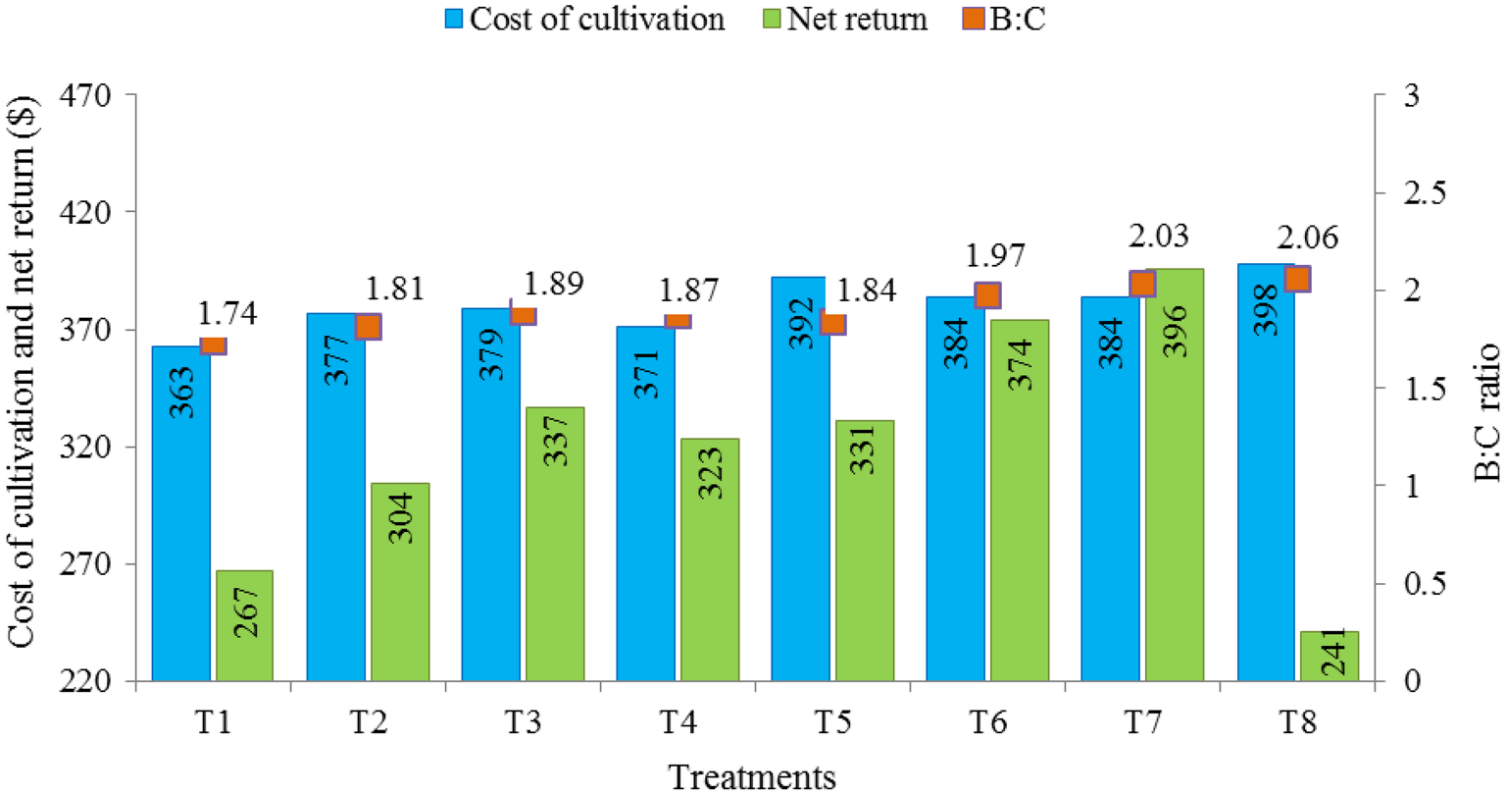
Effect of B, Zn and Fe biofortification on the cost of cultivation, net returns and economic analysis of mungbean. Treatment details are referred to in Table 1.

The economic analysis of mungbean cultivation as influenced by foliar use of borax, ZnSO_4_ and FeSO_4_ is shown in Fig. 1. The data indicated that the highest cost of cultivation was found for treatment T8 ($398) followed by treatment T7 ($384) and T6 ($384), whereas, the minimum cost of cultivation was observed in control ($363). The highest net return was recorded for treatment T8 ($421) followed by T7 ($396). Also, the B:C was the maximum recorded in treatment T8 (2.06) followed by T7 (2.03) and least in treatment T1 (1.74).

## Discussion

The results of the present study revealed the merits of the biofortification procedure in increasing the yield, concentration and uptake of micronutrients through foliar application of Zn, Fe and B in mungbean. Additionally, the combined foliar spray of Zn, Fe and B possessed economically superior outcomes based on higher net return and B:C ratio as compared to the other treatments. The results of different parameters are discussed in the following sections.

### Grain and straw yield with B, Zn and Fe application

Foliar application of ZnSO_4_. 7H_2_O (0.5%) + FeSO_4_. 7H_2_O (0.5%) + borax (0.1%) was proved to be effective for improving the grain and straw yield in mungbean (Table 2). This might be due to the synergistic interactions among all three nutrients i.e. B, Zn and Fe^28^. The enhancement in yield of grain and straw in the presence of B might be because of its involvement in elongation and cell division along with biomass accumulation which increased the yield^29^. Qamar et al.^30^ also reported similar findings where the use of B enhanced the yield in mungbean. Also, B might play a crucial role in photosynthetic as well as metabolic activities that resulted in improved yield^31^. A positive effect of B in the present study was reliable with the studies of other researchers in the case of rice^32^ and mungbean^30^. Similarly, an increase in grain and straw yield in mungbean was observed with the Zn foliar application which might be due to its role in photosynthesis, division of the cell, synthesis of protein, retention of membrane structure along with the ability to provide resistance against pathogen^23^. Another reason for increased yield might be related to the availability of Zn which helps in the synthesis of carbohydrates, lipids, protein as well as nucleic acid^33^ as they are considered crucial for the proper growth and development of the plant. Indeed, Zn also plays role in the formation of chlorophyll^34^ and this has been confirmed by several studies which explain the importance of Zn in the function of pollen, fertilization as well as germination^35,36^ which automatically leads to enhanced crop performance^37^. Furthermore, the foliar application of Fe also resulted in increased grain and straw yield, which might be attributed to the improved carbohydrate and protein synthesis as well as photosynthesis rate. Also, Fe has a crucial role in the synthesis of growth promoters like auxins, seed maturation, nucleic acid metabolism and chlorophyll synthesis which significantly results in higher grain and straw yield^38,39^. Foliar spray of Fe resulted in the higher translocation of photosynthates in reproductive structures which led to the increased number of effective branching, test weight and ultimately the grain and straw yield of mungbean^40^.

Additionally, the double and triple micronutrients application exhibited superior grain and straw yield over single micronutrients which might be due to the synergistic interactions involved among B, Zn and Fe. The results of the present study are supported by the above explanation that treatment T8 involving the use of all three micronutrients exhibited maximum yield of grain as well as straw in comparison to double micronutrient treatments i.e. T5, T6 and T7. Similarly, some studies reported that the joint use of Zn and B exhibited a higher impact on the yield of mungbean as compared to their sole application^41,42^. Furthermore, the study by Ali et al.^43^ presented that the joint use of B along with Mo or Zn leads to higher seed yield as compared to sole applications of B, Mo, or Zn.

### Boron, zinc and iron concentration in mungbean

The sole and combined application of B, Zn and Fe led to the increase in micronutrient concentration in mungbean grain and straw as compared to the control which might be due to the immediate absorption of available micronutrients by plant leaves^44^. Foliar application of Zn enhanced grain and straw Zn concentrations which is an outstanding method to produce grains with an adequate quantity of Zn. This approach would surely help in reducing malnutrition owing to Zn deficiency. A study demonstrated the potential of Zn in enhancing its concentration in the grain of mungbean^45^. Similar results were observed for the concentration of Fe in grain and straw of mungbean^46^. Increased Fe concentration in straw in comparison to grain might be associated with the presence of Fe storage proteins and non-heme proteins, which possess a good binding capacity for Fe. So, combined B, Zn and Fe application in the present study exhibited a positive influence on B, Zn and Fe content of mungbean grain and straw thus it can be inferred that B, Zn and Fe possess a similar mechanism for translocation to grains^47^. The enhancement in nutrient content might be due to an increased absorption as well as assimilation of the micronutrients that resulted in balanced nutritional value in the crop for higher growth and thereby higher nutrient content^48^.

### Boron, zinc and iron uptake

The results of the present study demonstrated that micronutrient uptake was found to increase significantly with external supplementation. The trend can be coupled with the joint impact of yield as well as concentration. Moreover, an exogenous supply of nutrients through different fertilizers resulted in higher nutrient availability as compared to the control. The results in the present study are in agreement with previous studies in which B and Fe application resulted in improved B and Fe uptake in mungbean^11,49^. Also, Zn possessed many important roles in plant including the formation of auxin and dehydrogenase enzyme activation^50^. It also helped in stabilizing the ribosomal fractions which increased the cation exchange capacity in roots, further helping in the formation of chlorophyll, regulation of auxin concentration, production of photosynthates along with their translocation to various parts of the plant including seeds that might result in absorption of increased quantity of the micronutrients from soil as well as improved concentration and uptake of these nutrients in the grain and straw of mungbean^51,52^. Overall, the combined application of B, Zn and Fe was found most effective to increase the micronutrient uptake in grain and straw^53^.

### Efficiency indices and economic analysis

The agronomic efficiency reflects the impact of fertilizer applied on economic returns. The trend suggested that the presence of B in the form of borax (0.1%) enhanced the mungbean production as compared to the ZnSO_4_.7H_2_O (0.5%) and FeSO_4_.7H_2_O (0.5%) alone. Moreover, the values were higher for MEI-Zn as compared to MEI-B and MEI-Fe which suggested higher mobility of Zn as compared to B and Fe. Additionally, the ARE measured the extent of nutrient loss from the cropping system and the effectiveness of management practices. In the present study, foliar application of micronutrients viz., Zn, Fe and B helped in overcoming the nutrient losses. Also, the results of PE indicated an increase in grain production with the absorbed nutrient. The higher values for PE-B, PE-Zn and PE-Fe were found in the treatment involving borax (0.1%) as compared to the treatment in which ZnSO_4_7H_2_O (0.5%) and FeSO_4_.7H_2_O (7H_2_O) were applied. Boron has multiple roles in plant physiology and the improved physiological efficiency through the foliar application of B can be easily seen in the present study as well as several earlier findings also^54,55^.

The cultivation cost, net return and B:C were affected positively through the use of B, Zn and Fe. Thus, the foliar application of ZnSO_4_.7H_2_O, FeSO_4_.7H_2_O and borax improved the economic outcomes of mungbean cultivation. The results are in agreement with the previous studies in which B application resulted in an enhanced B:C ratio of mungbean cultivation^49,56^. Also, the combined application of ZnSO_4_.7H_2_O (0.5%) + FeSO_4_.7H_2_O (0.5%) + borax (0.1%) exhibited greater net return and B:C ratio which proves its effectiveness over the sole application of micronutrients.

## Conclusions

Boron, zinc and iron are considered essential micronutrients in human body. Mungbean is an essential short-duration legume crop which can retain and enhance the productivity and nutrient quality of the crop through biofortification. The present study clarified that the supplementation of B, Zn and Fe through Borax, ZnSO_4_.7H_2_O and FeSO_4_.7H_2_O influenced the yield and quality of mungbean. The combined foliar spray of ZnSO_4_.7H_2_O (0.5%) + FeSO_4_. 7H_2_O (0.5%) + borax (0.1%) resulted in increased yield, micronutrient concentration and uptake in mungbean. The above treatment also possessed economically superior outcomes based on higher net return and B:C ratio as compared to the other treatments. Among the sole application of micronutrients, ZnSO_4_.7H_2_O (0.5%) treatment showed better results as compared to treatments involving FeSO_4_.7H_2_O and borax alone. Thus, the findings of the present study demonstrated the use of biofortification through the combined application of ZnSO_4_.7H_2_O (0.5%) + FeSO_4_. 7H_2_O (0.5%) + borax (0.1%) could be considered the most effective combination for enhancing yield, nutritional quality and economic returns of mungbean.

Guidelines and regulations by including a statement in the Methods section: It is certified that all methods were performed according to the relevant guidelines and regulations.

## Data Availability

The data will be available as per request to the corresponding author(s).

## References

[1] Majeed A, Minhas WA, Mehboob N, Farooq S, Hussain M, Alam S, Rizwan MS (2020). Iron application improves yield, economic returns and grain-Fe concentration of mungbean. PLoS ONE.

[2] Mehta, N., Rao, P. & Saini, R. Exploration of the antibacterial, antioxidant and anticancer potential of the seed coat extract of mungbean (Vigna radiata L. Wilczek). *Plant Archiv.* **21**(1), 1628–1633 (2021).

[3] Biswas, J.C., Kalra, N., Maniruzzaman, M., Choudhury, A.K., Jahan, M.A.H.S., Hossain, M.B., Ishtiaque, S., Haque, M.M. & Kabir, W. Development of mungbean model (MungGro) and its application for climate change impact analysis in Bangladesh. *Ecol. Model.* 384, 1–9 (2018).

[4] Singh, C.M., Singh, P., Tiwari, C., Purwar, S., Kumar, M., Pratap, A., Singh, S., Chugh, V. & Mishra, A.K.Improving drought tolerance in mungbean (Vigna radiata L. Wilczek): morpho-physiological, biochemical and molecular perspectives. *Agronomy* **11**(8), 1534 (2021).

[5] Kumar S, Ayachit G, Sahoo L (2020). Screening of mungbean for drought tolerance and transcriptome profiling between drought-tolerant and susceptible genotype in response to drought stress. Plant Physiol. Biochem..

[6] Kihara J, Bolo P, Kinyua M, Rurinda J, Piikki K (2020). Micronutrient deficiencies in African soils and the human nutritional nexus: opportunities with staple crops. Environ. Geochem. Health.

[7] Dhaliwal SS, Sharma V, Shukla AK (2022). Impact of micronutrients in mitigation of abiotic stresses in soils and plants-A progressive step toward crop security and nutritional quality. Adv. Agron..

[8] Zewide I, Sherefu A (2021). Review paper on effect of micronutrients for crop production. J. Nutr. Food Process.

[9] Zimicz, C. & Moretto, A. Boron toxicity on germination and early seedling growth of mung bean [*Vigna radiate* (L.) Wilczek] cv. Crystal. *Agronomía Ambiente* **39**, 26–32 (2019).

[10] Bautista-Diaz J, Cruz-Alvarez O, Hernández-Rodríguez OA, Sánchez-Chávez E, Jacobo-Cuellar JL, Preciado-Rangel P, Ojeda-Barrios DL (2021). Zinc sulphate or zinc nanoparticle applications to leaves of green beans. Folia Horticul..

[11] Jamal A, Khan MI, Tariq M, Fawad M (2018). Response of mung bean crop to different levels of applied iron and Zn. J. Horticul. Plant Res..

[12] Distéfano AM, López GA, Setzes N, Marchetti F, Cainzos M, Cascallares M, Zabaleta E, Pagnussat GC (2021). Ferroptosis in plants: triggers, proposed mechanisms, and the role of iron in modulating cell death. J. Exp. Bot..

[13] Chen WJ, Kung GP, Gnana-Prakasam JP (2022). Role of iron in aging related diseases. Antioxidants.

[14] Semba RD (2016). The rise and fall of protein malnutrition in global health. Ann. Nutr. Metab..

[15] Wu SH, Ho CT, Nah SL, Chau CF (2014). Global hunger: A challenge to agricultural, food, and nutritional sciences. Crit. Rev. Food Sci. Nutr..

[16] Vasconcelos MW, Gruissem W, Bhullar NK (2017). Iron biofortification in the 21st century: Setting realistic targets, overcoming obstacles, and new strategies for healthy nutrition. Curr. Opin. Biotechnol..

[17] Dissanayaka DMSB, Rankoth LM, Gunathilaka WMND, Prasantha BDR, Marambe B (2021). Utilizing food legumes to achieve iron and zinc nutritional security under changing climate. J. Crop Improv..

[18] Olson R, Gavin-Smith B, Ferraboschi C, Kraemer K (2021). Food fortification: The advantages, disadvantages and lessons from sight and life programs. Nutr..

[19] Dhaliwal SS (2022). Biofortification-A frontier novel approach to enrich micronutrients in field crops to encounter the nutritional security. Molecules.

[20] Ramzan Y, Hafeez MB, Khan S, Nadeem M, Batool S, Ahmad J (2020). Biofortification with zinc and iron improves the grain quality and yield of wheat crop. Int. J. Plant Prod..

[21] Jalal, A., Shah, S., Teixeira Filho, M.C.M., Khan, A., Shah, T., Ilyas, M. & Rosa, P.A.L. Agro-biofortification of zinc and iron in wheat grains*. Gesunde Pflanzen,* **72**(3), 227–236 (2020)*.*

[22] Aziz, M. Z., Yaseen, M., Abbas, T., Naveed, M., Mustafa, A., Hamid, Y., Saeed, Q. & XU, M. Foliar application of micronutrients enhances crop stand, yield and the biofortification essential for human health of different wheat cultivars. *J. Integr. Agric.* **18**, 1369–1378 (2019).

[23] Kanwal A, Khan MB, Hussain M, Naeem M, Rizwan MS, Zafar-ul-Hye M (2020). Basal application of zinc to improve mung bean yield and zinc-grains-biofortification. Phyton-Int. J. Exp. Bot..

[24] Haider, M. U., Hussain, M., Farooq, M. & Nawaz, A. Optimizing zinc seed priming for improving the growth, yield and grain biofortification of mungbean (*Vigna radiate* (L.) wilczek). *J. Plant Nutr*. 43, 1438–1446 (2020).

[25] Farooq U (2021). Biofortification of mungbean (*Vigna radiata*) using iron-enriched organic amendment. Pak. J. Agric. Res..

[26] Kumar, B. & Dhaliwal, S. S. Zinc biofortification of dual purpose cowpea [*Vigna unguiculata* (L.) Walp.] for enhancing productivity and nutritional quality in a semi-arid region of India. *Arch. Agron. Soil Sci.* **68**, 1034–1048 (2021).

[27] Dhaliwal, S. S. *et al.* Assessment of agroeconomic indicators of *Sesamum indicum* L. as influenced by application of boron at different levels and plant growth stages. *Molecules 26*, 6699 (2021). 10.3390/molecules26216699 PMC8587077 34771108

[28] Soni, J. & Kushwaha, H.S. Effect of foliar spray of zinc and iron on productivity of mungbean [*Vigna radiata* (L.) Wilczeck]. *J. Pharmacogn. Phytochem.* **9**(1), 108–111 (2020).

[29] Mamatha K, Vidyasagar GECh, Laxminarayana P, Padmaja G (2017). Effect of boron levels and farmyard manure on physiological growth and quality of sesame (*Sesamum indicum*). Int. J. Curr. Microbiol. Appl. Sci..

[30] Qamar J, Rehman A, Ali MA, Qamar R, Ahmed K, Raza W (2016). Boron increases the growth and yield of mungbean. J. Adv. Agric..

[31] Simkin AJ, Lopez-Calcagno PE, Raines CA (2019). Feeding the world: Improving photosynthetic efficiency for sustainable crop production. J. Exp. Bot..

[32] Atique-ur-Rehman, Farooq, M., Rashid, A., Nadeem, F., Stuerz, S., Asch, F., Bell, R. W. & Siddique, K. H. M. Boron nutrition of rice in different production systems. A review. *Agron. Sustain. Develop.* **38**, 25 (2018).

[33] Minnocci, A., Francini, A., Romeo, S., Sgrignuoli, A. D., Povero, G. & Sebastiani, L. Zn-localization and anatomical changes in leaf tissues of green beans (*Phaseolus vulgaris* L.) following foliar application of Zn-lignosulfonate and ZnEDTA. *Sci. Hortic.* **231**, 15–21 (2018).

[34] Shalal, K. H. & Mohammed, H.A. The effect of zinc and abscisic acid on the growth of mung bean plant affected by moisture tension. *Ann. R.S.C.B.* **25**, 135–151 (2021).

[35] Umair Hassan, M., Aamer, M., Umer Chattha, M., Haiying, T., Shahzad, B., Barbanti, L., Nawaz, M., Rasheed, A., Afzal, A., Liu, Y. & Guoqin, H. The critical role of zinc in plants facing the drought stress. *Agriculture* **10**(9), 396 (2020).

[36] Alwahibi, M.S., Elshikh, M.S., Alkahtani, J., Muhammad, A., Khalid, S., Ahmad, M., Khan, N., Ullah, S. and Ali, I. Phosphorus and zinc fertilization improve zinc biofortification in grains and straw of coarse vs. fine rice genotypes. *Agronomy,* **10**(8), 1155 (2020).

[37] Shojaei, H. & Makarian, H. The effect of nano and non-nano zinc oxide particles foliar application on yield and yield components of mungbean (*Vigna radiate* L.) under drought stress. *Iranian J. Field Crop. Res.* **12**, 727–737 (2015).

[38] Pal, V., Singh, G. & Dhaliwal, S. S. Yield enhancement and biofortification of chickpea *Cicer arietinum L.* grain with iron and zinc through foliar application of ferrous sulfate and urea. *J. Plant Nutr.* **42**, 1789–1802 (2019).

[39] Schmidt W, Thomine S, Buckhout TJ (2020). Iron nutrition and interactions in plants. Front. Plant Sci..

[40] Saini AK, Singh R (2017). Effect of Sulphur and iron fertilization on growth and yield of greengram. Int. J. Curr. Microbiol. Appl. Sci..

[41] Islam MS, Hasan K, Sarkar NA, Sabagh AE, Rashwan E, Barutcular C (2017). Yield and yield contributing characters of mungbean as influenced by zinc and boron. Agric. Adv..

[42] Alam MS, Islam MF (2016). Effect of zinc and boron on seed yield and yield contributing traits of mungbean in acidic soil. J. Biosci. Agr. Res..

[43] Ali, M.M., Anwar, R., Shafique, M.W., Yousef, A.F. & Chen, F. Exogenous application of Mg, Zn and B influences phyto-nutritional composition of leaves and fruits of loquat (*Eriobotrya japonica* Lindl.). *Agronomy* **11**(2), 224 (2021).

[44] Suganya A, Saravanan A, Manivannan N (2020). Role of zinc nutrition for increasing zinc availability, uptake, yield, and quality of maize (*Zea mays* L.) grains: An overview. Commun. Soil Sci. Plant Anal..

[45] Haider MU, Hussain M, Farooq M, Nawaz A (2018). Soil application of zinc improves the growth, yield and grain zinc biofortification of mungbean. Soil Environ..

[46] Jamal A, Khan MI, Tariq M, Fawad M (2018). Response of mung bean crop to different levels of applied iron and zinc. J. Hortic. Plant Res..

[47] Kawakami Y, Bhullar NK (2018). Molecular processes in iron and zinc homeostasis and their modulation for biofortification in rice. J. Integr. Plant Biol..

[48] Zewail RMY, El-Gmal IS, Khaitov B, El-Desouky HS (2020). Micronutrients through foliar application enhance growth, yield and quality of sugar beet (*Beta vulgaris* L.). J. Plant Nutr..

[49] Quddus MA (2020). Impact of zinc, boron and molybdenum addition in soil on mungbean productivity, nutrient uptake and economics. J. Agric. Sci..

[50] Rudani L, Vishal P, Kalavati P (2018). The importance of zinc in plant growth-A review. Int Res. J. Nat. Appl. Sci..

[51] Khan, S.T., Malik, A. & Ahmad, F. Role of Zinc Homeostasis in Plant Growth. In: Khan, S.T., Malik, A. (eds) *Microbial Biofertilizers and Micronutrient Availability* (Springer, Cham, 2022). 10.1007/978-3-030-76609-2_9

[52] Gahlot N, Singh U, Ram M, Mehriya ML, Borana H, Mandiwal M (2020). Biochemical assessment and yield of mungbean as influenced by zinc and iron fertilization. Chem. Sci. Rev. Lett..

[53] Dhaliwal SS (2021). Interactive effect of foliar application of nitrogen, zinc and iron on productivity and oil nutritional quality of Indian mustard (*Brassica juncea* L.). Agron..

[54] Abdel-Motagally FMF, El-Zohri M (2018). Improvement of wheat yield grown under drought stress by boron foliar application at different growth stages. J. Saudi Soc. Agric. Sci..

[55] Botelho, R.V., Müller, M.M.L., Umburanas, R.C., Laconski, J.M.O. & Terra, M.M. Boron in fruit crops: plant physiology, deficiency, toxicity, and sources for fertilization. In *Boron in Plants and Agriculture* (pp. 29–50). Academic Press (2022).

[56] Mubeen A, Saeed MT, Saleem MF, Wahid MA (2020). Zinc and boron application improves yield, yield components and gross returns of mungbean (*Vigna radiata* L.). J. Arable Crops Market..

